# Antiplasmodial activity and cytotoxicity of ethanol extract of *Zea mays* root

**Published:** 2017

**Authors:** Jude Efiom Okokon, Bassey Sunday Antia, Bala Adamu Azare, Patience Jude Okokon

**Affiliations:** 1 *Department of Pharmacology and Toxicology, Faculty of Pharmacy, University of Uyo, Uyo, Nigeria *; 2 *Department of Chemistry, University of Uyo, Uyo, Nigeria*; 3 *Department of Zoology, University of Abuja, Abuja, Nigeria*; 4 *Department of Clinical Pharmacy and Biopharmacy, Faculty of Pharmacy, University of Uyo, Uyo, Nigeria*

**Keywords:** Antimalarial, Antiplasmodial, Plasmodium falciparum, P. berghei, Zea mays

## Abstract

**Objective:**

*Zea mays *root decoction that has been traditionally used for the treatment of malaria by various tribes in Nigeria, was evaluated for antimalarial potential against malaria parasites using *in vivo* and *in vitro* models.

**Materials and Methods:**

The root extract of *Zea mays *was investigated for antimalarial activity against *Plasmodium berghei *in mice using rodent malaria models; suppressive, prophylactic and curative tests and *in vitro* antiplasmodial activity against chloroquine-sensitive (Pf 3D7) and resistant (Pf INDO) strains of *Plasmodium falciparum* using SYBR green assay method. Median lethal dose and cytotoxic activity against HeLa and HEKS cells were assessed and phytochemical screening was also carried out using standard procedures.

**Results:**

The LD_50_ value of root extract was found to be 474.34 mg/kg. The crude extract (45-135 mg/kg, p.o) showed significant (p<0.05-0.001) antimalarial activity against *P. berghei* infection in suppressive, prophylactic and curative tests with a prolonged survival time. The crude extract also showed moderate activity against both chloroquine-sensitive (Pf 3D7) and resistant (Pf INDO) strains of *P. falciparum* with an IC_50_ value of 71.62±3.38 μg/ml (for Pf 3D7) and 63.76±4.12 μg/ml (for Pf INDO). The crude extract was not cytotoxic to the two cell lines tested with TC_50_ of >100 μg/ml against both HeLa and HEKS cell lines.

**Conclusion:**

These results suggest that the root extract of *Zea mays *possesses antimalarial activity against both chloroquine-sensitive and resistant malaria and these data justify its use in ethnomedicine to treat malaria infections.

## Introduction

*Zea mays* L. (Family Poaceae), known as maize or corn, is an annual grass plant cultivated for human consumption and rearing of animals. It was introduced to Nigeria in the 16th century (Osagie and Eka, 1998 ▶). The plant has tall, erect stalk with long leaves and bears ears that are enclosed in the husks, which are modified leaves (Simmonds, 1979 ▶). It also has a fibrous root system. Besides its nutritive values, maize grains, leaves, corn silks, stalk, and inflorescence are also used in ethnomedicine for the treatment of several ailments. The corn silk is used as an antidiabetic diuretic, and decoction of the silk is consumed for the treatment of urinary problems and gallstones (Foster and Duke, 1990 ▶; Gill, 1992 ▶; Abo etal., 2008 ▶). The ash of the cob is used for the treatment of cough (Gill, 1992 ▶) as well as inflammatory diseases. The husks are used in the treatment of pains and arthritis (Owoyele et al., 2010 ▶). It is also taken as warm tea for the treatment of malaria in Ibibio traditional medicine. Biological activities reported on the leaf extract include anticancer (Balasubramanian et al., 2014 ▶), antioxidant (Balasubramanian and Padma, 2012 ▶) and antioxidative stress (Balasubramanian and Padma, 2013 ▶; Balasubramanian et al., 2015 ▶) activities. Anti-inflammatory and analgesic activities have been reported on the husk extract (Owoyele et al., 2010 ▶). Eight phenolic compounds (gallic acid, protocatechuic acid, chlorogenic acid, cafeic acid, femlic acid, rutin, resveratrol, and kaempferol) have also been detected in ethanol extract of *Z. mays* husk (Dong et al., 2014 ▶). Phytochemical compounds with antifungal activity such as 6-methoxybenzoxazolinone and 6,7-dimethoxybenzoxazolinone, and (6R)-7,8-dihydro- 3-oxo-α-ionone and (6R; 9R)-7,8-dihydro-3-oxo- α -ionol were isolated from root extract and root exudates of *Z. mays* (Park et al., 2004 ▶).

Information on the biological activities of the root extract is scarce. In this study, we report the antimalarial and antiplasmodial activities of *Z. mays* root extract to confirm its use in Ibibio ethnomedicine.

## Materials and Methods

**Animals**

The animals (Swiss albino mice) of either sex were used for these experiments. The animals were housed in standard cages and they had free access to standard pelleted feed (Guinea feed) and water, *ad libitum*. Permission and approval for animal studies were obtained from the College of Health Sciences Animal Ethics Committee, University of Uyo.

**Parasite used**

A chloroquine-sensitive strain of *Plasmodium berghei* (ANKA) was obtained from the National Institute of Medical Research (NIMER), Yaba, Lagos, Nigeria and maintained by sub-passage in mice. While *P. falciparum;* chloroquine-sensitive (Pf 3D7) and resistant (Pf INDO) strains were obtained from the International Center for Genetic Engineering and Biotechnology, New Delhi, India.

**Collection of plant materials**

Fresh roots of *Z. mays* were collected in August 2015 from Farmland in Uyo LGA, Akwa Ibom State, Nigeria. The roots were identified and authenticated as *Z. mays* by Dr. Margaret Bassey, a taxonomist from the Department of Botany and Ecological studies, University of Uyo, Uyo, Nigeria. Herbarium specimen was deposited at the Faculty of Pharmacy Herbarium, University of Uyo, Uyo (FPH 614).

**Extraction**

The plant parts (roots) were washed and air-dried on laboratory table for 2 weeks. The dried roots were pulverized using a pestle and mortar. The powdered root was macerated in 95% ethanol for 72 hr. The liquid ethanol extract obtained by filtration was evaporated to dryness in a water bath at 60°C. The yield of the extract was stored in a refrigerator at -4°C until it was used for the experiments.

**Phytochemical screening**

Phytochemical screening of the crude root extract was carried out employing standard procedures and tests (Trease and Evans, 1996 ▶, Sofowora, 1993 ▶).

**Determination of median lethal dose (LD**_50_ **)**

 The median lethal dose (LD_50_) of the extract was estimated using albino mice by intraperitoneal (i.p) route using the method of Lorke’s (1983) ▶. This involved intraperitoneal administration of different doses of the extract (1000 – 5000 mg/kg) to groups of five mice each. The animals were observed for manifestation of physical signs of toxicity such as writhing, decreased motor activity, decreased body/limb tone, decreased respiration and death. The number of deaths in each group within 24 hours was recorded.

**Parasite inoculation**

Each mouse used in the experiment was inoculated intraperitoneally with 0.2 ml of infected blood containing about 1 x 10^7 ^*P. berghei berghei *parasitized erythrocytes. The inoculum consisted of 5 x 10^7^ *P. berghei berghei* erythrocytes per ml. This was prepared by determining both the percentage parasitaemia and the erythrocytes count of the donor mouse and diluting the blood with isotonic saline in proportions indicated by both determinations (Odetola and Basir, 1980 ▶).

**Drug** **a** **dministration**

The drug (artesunate Na), and extract used in the antiplasmodial study were orally administered with the aid of a stainless metallic feeding cannula.

**Evaluation of anti-plasmodial activity of ethanol extract of** ***Zea mays*** **crude root**

Evaluation of suppressive activity of the extract (4-day test).

This test was used to evaluate the schizontocidal activity of the extract and artesunate against early *P. berghei *infection in mice. This was done as described by Knight and Peters (1980) ▶. Thirty mice were randomly divided into five groups of six mice each. On the first day (D_0_), the mice were infected with the parasite and randomly divided into groups. Animals were administered with the extract and artesunate. The mice in group 1 were administered with the 45 mg/kg, group 2 with 90 mg/kg and group 3 with 135 mg/kg of crude extract, while group 4 was administered with 5 mg/kg of artesunate Na (positive control), and 10 ml/kg of distilled water was given to group 5 (negative control) for four consecutive days (D_0_ – D_3_) between 8am and 9am. On the fifth day (D_4_), thin blood film was made from tail blood. The film was then stained with Leishman’s stain to reveal the number of parasitized erythrocytes out of 500 erythrocytes in a random field of the microscope. The average percentage of suppression of parasitaemia was calculated in comparison with the controls as follows:

Average % parasitaemia in negative control- Average % parasitaemia in positive groupsAverage % parasitaemia in negative control

**Evaluation of** **p** **rophylactic or** **r** **epository** **a** **ctivities of the** **e** **xtract**

The repository activity of the extract and artesunate Na was assessed using the method described by Peters (1965) ▶. The mice were randomly divided into seven groups of six mice each. Groups 1-3 were administered with 45, 90 and 135 mg/kg/day of the extract, respectively. Groups 4 and 5 were respectively administered with 5 mg/kg/day of artesunate (positive control) and 10 ml/kg of distilled water (negative control). Administration of the extract/drug was continued for three consecutive days (D_0_ – D_2_). On the fourth day (D_3_), the mice were inoculated with *P. berghei.* Seventy-two hours later, the parasitaemia level was assessed in blood smears.

**Evaluation of** **c** **urative** **a** **ctivities of the** **e** **xtract** **(Rane’s test)**

This was used to evaluate the schizontocidal activity of the extract, and artesunate in an established infection. This was done as described by Ryley and Peters (1970) ▶. *P. berghei *was injected intraperitoneally into another 30 mice on the first day (D_O_). Seventy–two hours later (D_3_), the mice were randomly divided into five groups of six mice each. The extract at the doses of 45 mg/kg, 90 mg/kg and 135 mg/kg was orally administered to groups 1-3, respectively. Also, 5 mg/kg/day of artesunate Na was administered to the group 4 (positive control) and group 5 was given 10 ml/kg of distilled water (negative control). The extract and drugs were administered once daily for 5 days. Leishman’s stained thin smears were prepared from tail blood samples collected on each day of treatment to monitor parasitaemia level. The mean survival time (MST) of the mice in each treatment group was determined over a period of 29 days (D_0_ – D_28_).

No. of days survived Total No. of days (29)×100=MST

**Evaluation of** ***in vitro*** **antiplasmodial activity**

*In vitro cultivation of Plasmodium falciparum*

CQ-sensitive strain 3D7 and CQ-resistant strain INDO of *Plasmodium falciparum* used in this study were *in vitro* blood stage culture to test the antimalarial efficacy of the crude root extract and fractions. The culture was maintained at Malaria Research Laboratory, International Centre for Genetic Engineering and Biotechnology, New Delhi, India. *P. falciparum *culture was maintained according to the method described by Trager and Jensen (1976) ▶ with minor modifications. *P. falciparum* (3D7) cultures were maintained in fresh O+ve human erythrocytes suspended at 4% hematocrit in RPMI 1640 (Sigma) containing 0.2% sodium bicarbonate, 0.5% albumax, 45 µg/L hypoxanthine, and 50 µg/L gentamicin and incubated at 37 ◦C under a gas mixture of 5% O_2_, 5% CO_2_, and 90% N_2_. Every day, infected erythrocytes were transferred into fresh complete medium to propagate the culture. For *P. falciparum* (INDO strain) in culture medium, albumax was replaced with 10% pooled human serum.

**Drug dilutions**

Stock solutions of the plant extract and fractions as well as artemisinin were prepared in dimethyl sulfoxide (DMSO), while CQ stock solution was prepared in water (Milli-Q grade). All stocks were then diluted with culture medium to achieve the required concentrations (in all cases except CQ, the final solution contained 0.4% DMSO, which was found to be nontoxic to the parasite). Drugs and test plant extracts were then placed in 96-well flat bottom tissue culture grade plates.

***In vitro*** **antiplasmodial assays**

The crude root extract and fractions of this plant were evaluated for their antimalarial activity against 3D7 and INDO strains of *P. falciparum*. For drug screening, SYBR green I-based fluorescence assay was set up as previously described (Smilkstein et al., 2004 ▶). Sorbitol-synchronized parasites were incubated under normal culture conditions at 2% hematocrit and 1% parasitemia in the absence or presence of increasing concentrations of the plant extract and fractions. CQ and artemisinin were used as positive controls, while 0.4% DMSO was used as the negative control. After 48 hr of incubation, 100 µl of SYBR Green I solution (0.2 µl of 10,000× SYBR Green I (Invitrogen)/mL) in lysis buffer[Tris (20 mM; pH 7.5), EDTA (5 mM), saponin (0.008%, w/v), and Triton X-100 (0.08%, v/v)] was added to each well and mixed twice gently with multi-channel pipette and incubated in dark at 37 ◦C for 1 hr. Fluorescence was measured with a Victor fluorescence multi-well plate reader (Perkin Elmer) with excitation and emission wavelengths of 485 and 530 nm, respectively. The fluorescence was plotted against the drug concentration and the 50% inhibitory concentration (IC_50_) was determined by analysis of dose–response curves. Results were microscopically validated by examination of Giemsa stained smears of extract-treated parasite cultures.

**Evaluation of cytotoxic activity against HeLa and HEKS cells using MTT assay**

The cytotoxic effects of the extract and fractions on host cells were assessed by functional assay as previously described (Mosmann, 1983 ▶) using HeLa cells cultured in RPMI containing 10% fetal bovine serum, 0.21% sodium bicarbonate (Sigma) and 50 µg/mL gentamicin (complete medium) and Human Embroynic kidney 293 cells cultured in DMEM and supplemented with 10% fetal Bovine albumin. 

Briefly, cells (10^4^ cells/200 µl/well) were seeded into 96-well flat-bottom tissue culture plates in complete medium. Then, drug solutions were added after 24 hr of seeding and incubated for 48 hr in a humidified atmosphere at 37 ◦C with 5% CO_2_. DMSO (as positive inhibitor) was added at 10%. Twenty microliters of a stock solution of MTT (5 mg/mL in 1X phosphate buffered saline) was added to each well, gently mixed and incubated for another 4 hr. After spinning the plate at 1500 rpm for 5 min, supernatant was removed and 100 µl of DMSO (stop agent) was added. Formation of formazan was evaluated using a microtiter plate reader (Versa max tunable multi-well plate reader) at 570 nm. The 50% cytotoxic concentration (TC_50_) of drug was determined by analysis of dose–response curves.

**Statistical analysis**

Data obtained from this work were statistically analyzed using Students’ t-test and ANOVA (One- or Two- way) followed by a post-test (Turkey-Kramer multiple comparison test). Differences between means was considered significant if p<0.05.

## Results

**Phytochemical Screening**

Phytochemical screening of the crude root extract revealed the presence of chemical constituents such as alkaloids, flavonoids, tannins, terpenes, saponins, cardiac glycosides.

**Determination of median lethal dose (LD**_50_ **)**

The Median Lethal Dose (LD_50_) was 474.34 mg/kg. The physical signs of toxicity included excitation, paw licking, increased respiratory rate, decreased motor activity, gasping and coma which was followed by death.

**E** **ffect** **o** **n suppressive activity** **o** **f ethanol extract** **o** **f** ***Zea mays*** **root**

The extract showed a dose-dependent chemosuppressive effect on parasitaemia. These effects were statistically significant as compared to the control (p<0.05 - 0.001). The chemoinhibitory percentages ranged from 69.18 to 88.72 (Table 1). However, the effect of the extract was incomparable to that of the standard drug, artesunate, with a chemosuppression of 98.82% (Table 1).

**Table 1:** Suppressive activity of Zea mays root extract (4-day test). Values are expressed as mean±SEM. Significance relative to control:cp<0.001 (n=6).

	**Dose **	**Parasitaemia**	**% chemosuppression**
**Distilled water**	10 ml/kg	44.33 ± 2.18	_
**Extract**	45 mg/kg	13.66 ±0.72^c^	69.18
	90 mg/kg	10.33 ± 2.40^c^	76.69
	135mg/kg	5.00 ± 1.52^c^	88.72
**Artesunate**	5 mg/kg	0.52 ± 0.01^c^	98.82

**Effect** **o** **n repository activity** **o** **f ethanol extract** **o** **f** ***Zea mays*** **root**

The ethanol extract of *Zea mays* root showed a dose-dependent chemosuppressive effect (6.82 – 65.89%) on the parasitaemia during prophylactic studies. These effects were statistically significant as compared to the control (p<0.001). However, these effects were lower compared to that of the standard drug, artesuante Na, with a chemosuppression of 90.92 % (Table 2).

**Antiplasmodial effect** **o** **f ethanolic extract** **o** **f** ***Zea mays*** **root** **on** **established infection**

The extract showed a dose- dependent schizonticidal effect on the parasitaemia. There were reductions in the percentage of parasitaemia of the extract/artesunate-treated groups compared to that of the control in which prominent increases were recorded. These reductions were statistically significant as compared to the control (p<0.05-0.001) (**Table 3**). Though the extract showed asignificant (p<0.05-0.001), dose-dependent mean survival time on established infection, the effect of the extract (45-135 mg/kg) was lower compared to that of the standard drug, artesunate (Table 4). 

**Figure 1:**
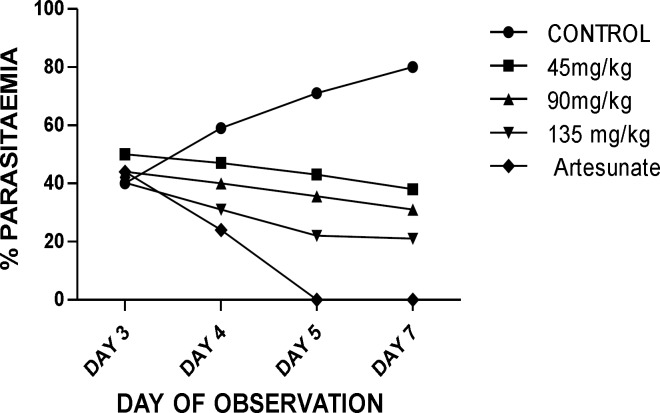
Antiplasmodial activity of root extract of Zea mays (curative test

***In vitro*** **antiplasmodial and cytotoxic activities**

The results of the *in vitro* studies show that the root extract exerted antiplasmodial activity against chloroquine-sensitive Pf 3D7 and resistant Pf INDO strains of *P. falciparum* (Table 4). The root extract moderate activity against both strains of *P. falciparum *with IC_50_ value of 71.62± 2.34 μg/ml (Pf 3D7) and 63.76±4.24 μg/ml (Pf INDO). The crude root extract was negligibly cytotoxic to the two cell lines tested with TC_50_ of >100 μg/ml against both HeLa and HEKS cell lines, respectively (Table 5).

**Table 2:** Repository/Prophylactic activity of ethanol extract of Zea mays root on Plasmodium berghei infection in mice. Values are expressed as mean ± SEM. Significance relative to control cp<0.001 (n=6).

	**Dose**	**Parasitaemia**	**% Chemosuppression**
**Normal saline**	10 ml/kg	14.66±0.66	-
**Crude extract**	45 mg/kg	13.66± 2.72	6.82
	90 mg/kg	10.33±2.40	29.53
	135 mg/kg	5.00 ±1.52^C^	65.89
**Artesunate**	5.0 mg/kg	1.33±0.66^c^	90.92

**Table 4:** Mean Survival Time (MST) of mice receiving different doses of root extract of Zea mays during established infection. Values are expressed as mean ±S.E.M. Significance relative to control: ap<0.05; p<0.001 (n=6)

	**Dose**	**MST (days)**
**Distilled water**	10ml/kg	14.33±0.33
**Extract**	45 mg/kg	14.66 ± 0.66
	90 mg/kg	17.38 ±1.20^a^
	135 mg/kg	25.66± 1.33^ c^
**Artesunate**	5 mg/kg	30.00 ± 0.00^ c^

**Table 5:** In vitro antiplasmodial and cytotoxic activities of crude root extract of Zea mays

	**IC50(µg/m)** ***Pf*** ** 3D7**	**IC50(µg/ml)** *** Pf*** ** INDO**	**Cytotoxicity**	
			**Hela cells** **TC**_50_ **(µg/ml)**	**HEKS cells** **TC**_50_ **(µg/ml)**
**Crude extract**	71.62±3.38	63.76 ± 4.12	>100	>100
**Chloroquine**	0.021	0.258	>200	-
**Artemisinin**	0.0045	0.0045	>200	-

## Discussion

*Zea mays *root decoctions are traditionally used as malaria remedy and febrifuge by various tribes in Nigeria to treat malaria infections and fever associated with the diseases. This work was undertaken to investigate the antiplasmodial potential of the root extract to provide scientific basis to these claims.

In this work, median lethal dose (LD_50_) was determined to be 474.34 mg/kg, and the extract was found to be relatively safe with moderate toxicity (Homburger, 1989 ▶).

The antiplasmodial activity of root extract of *Zea mays *was also investigated using standard models. It was found that the extract significantly reduced the parasitaemia in prophylactic, suppressive and curative models in a dose-dependent fashion. However, the root extract only exerted moderate activity against chloroquine-sensitive *P. falciparum* (3D7) strain and chloroquine-resistant strain (INDO). The variability in the activity of the root extract in both *in vivo* and *in vitro* studies, suggests the involvement of immune system in the activity of the root extract which could probably be immunostimulation. It further suggests that this plant may either be an immune stimulant or it may alleviate the symptoms of malaria such as pains and fever among others. Kirby (1997) ▶ had reported that some plants that are locally used as malarial remedies may only alleviate the symptoms associated with malaria without having any significant effect on the parasites as shown in this study (Kirby, 1997 ▶).

Moreso, plant compounds that suppress or partly inhibit the growth of the parasite (plasmodistatic) as well as those that stimulate the immune system or provide symptomatic cure and reverse some pathological features of malaria infection are reported to potentiate malaria resistance and antiplasmodial activity in immune individuals living in endemic areas (Kirby, 1997 ▶). Therefore, this plant may help the immune system to develop resistance to malaria and in a way, antimalarial activity.

Some secondary metabolites of plants such as alkaloids, terpenes and flavonoids have been reported to have antiplasmodial activity (Philipson and Wright,1991 ▶; Christensen and Kharazmi,2001 ▶; Kirby et al., 1989 ▶). These compounds were found to be present in the extract studied and may be responsible for the observed antiplasmodial activity of the extract, though the active principle is yet to be identified. Although the mechanism of action of this extract has not been elucidated, flavonoids are known to exert antiplasmodial activity by chelating with nucleic acid base pairing of the parasite (Lui et al., 1992 ▶), thereby producing plasmocidal effects. Other modes of action of flavonoids include modulation of host immunity to tackle disease and inhibition of plasmodial enoyl-ACP reductase (FAB I enzyme), a key regulator of type II fatty synthases (FAS-II) in *P. falciparum* (Teffo et al., 2010 ▶; Kirmizibekmez et al., 2004 ▶). Flavonoids may also bind parasite’s serine/threonine kinase with high affinity and affect its development (Ferreira et al., 2010 ▶). Also, some plants are known to exert antiplasmodial action either by causing elevation of red blood cell oxidation (Etkin, 1997) or by inhibiting protein synthesis (Kirby et al., 1989 ▶). The extract may be acting through one of these mechanisms to exert antiplasmodial activity observed in this study.

Maize plant is known as a rich source of phenolics (Dong et al., 2014 ▶) with antioxidant potentials. Some of these phenolic compounds like gallic acid and rutin present in *Zea mays* parts, have been reported to possess antimalarial activity (Horgen et al., 1997 ▶; Attioua et al., 2011 ▶). Rutin has been shown to possess significant antiplasmodial activity against chloroquine-sensitive and resistant strains of *P. falciparum *with IC_50_ of 3.53±13.34 µM against 3D7 and 15.00 µM against K1 (Attioua et al., 2011 ▶). 

Besides, antioxidant potentials of some plant and natural products especially flavonoids have been found to promote schizoniticide activity by modulating the cellular signaling pathway (Al-Adhroey et al., 2011 ▶) and this has been suggested to be responsible for antiplasmodial activity of compounds such as quercetin (Cimanga et al., 2009 ▶; Ganesh et al., 2012 ▶), as elevated free radicals levels are common features of malaria disease and are implicated in severe malaria complications. This could be one of the modes of action of this extract as it contains antioxidant phenolics and flavonoids. 6-methoxybenzoxazolinone, 6, 7-dimethoxybenzoxazolinone, (6R)-7,8-dihydro- 3-oxo-α-ionone and (6R; 9R)-7,8-dihydro-3-oxo- α -ionol have been isolated from the root extract and exudates of *Zea mays* (Park et al., 2004 ▶). These compounds from the root may likely possess antiplasmodial activity.

In this study, the root extract of *Zea mays* was also found to contain terpenes among others. Terpenes and their derivatives such as monoterpenes and sesquiterpenes have been implicated in antiplasmodial activity of many plants (Philipson and Wright, 1991 ▶; Christensen and Kharazmi, 2001 ▶). Monoterpenes such as limonene have been implicated in endoperoxidation leading to plasmocidal activity (Hatzakis et al., 2000 ▶). These may also contribute to the antiplasmodial activity of this extract.

The result obtained in this study indicated that the root of *Zea mays* plant possesses a significant antiplasmodial activity in *in vivo* and *in vitro* models which justifies the usage of this plant in the treatment of malaria.
